# A focus group interview with health professionals: establishing efficient transition care plan for older adult patients in Korea

**DOI:** 10.1186/s12913-022-07802-z

**Published:** 2022-03-26

**Authors:** Chan Mi Park, Seung Jun Han, Jae Hyun Lee, Jin Lim, Sung do Moon, Hongran Moon, Seo-Young Lee, Hyeanji Kim, Il-Young Jang, Hee-Won Jung

**Affiliations:** 1 Harvard T.H. Chan School of Public Health, MA Boston, USA; 2 Department of Internal Medicine, Seoul National University Hospital, 101 Daehak-ro, Jongno-gu, Seoul, 03080 Korea; 3 Hospital Medicine Center, Seoul National University Hospital, Seoul, Korea; 4 Regional Emergency Medical Center, Seoul National University Hospital, Seoul, Korea; 5 Division of Geriatrics, Department of Internal Medicine, Asan Medical Center, University of Ulsan College of Medicine, 88, Olympic-ro 43-gil, Seoul, 05556 Songpa-gu Korea

**Keywords:** Patient transfer, Transitional care, Hospitalization, Patient care plan

## Abstract

**Background:**

Although transition care planning can affect the functional status and quality of life after acute hospitalization in older adults, little is known on problems associated with discharge planning in acute care hospitals in Korea. We aimed to investigate barriers and possible solutions on transfer planning of complex older patients in this study.

**Methods:**

We used focus group interviews with the application of framework analysis. Twelve physicians providing inpatient care from 6 different institutions in Korea participated in the interview. Facilitating questions were extracted from 2 roundtable meetings prior to the primary interview. From transcribed verbatim, themes were constructed from corresponding remarks by participants.

**Results:**

We revealed two main domains of the barrier, which included multiple subdomains for each of them. The first domain was a patient factor barrier, a composite of misperception of medical providers’ intentions, incomprehension of the healthcare system, and communication failure between the caregivers or decision-makers. The second domain, institutional factors included different fee structures across the different levels of care, high barrier to accessing health service in tertiary hospitals or to be referred to, the hardship of communication between institutions, and insufficient subacute rehabilitation service across the country.

**Conclusions:**

Through the interview, physicians in the field recognized barriers to a smooth transition care process from tertiary level hospitals to community care, especially for older adults. Participants emphasized both the patients and hospital sides of adjustment on behaviors, communication, and greater attention for the individuals during the transition period.

## Background

As the older population with complex care needs with multiple acute and/or chronic diseases is growing, it is inevitable to have a disproportionately higher rate of hospitalization experience for older adults than the younger population. In older patients, functional care needs commonly coexist on top of multimorbidity, and their functional status dynamically interacts with underlying illness leading to adverse effects by hospitalization [1]. Therefore, the transitional care plan is critical to ensure patients are discharged from the hospital to an appropriate place to improve clinical and functional outcomes [2]. Previous studies showed that strategic discharge planning and transitional care interventions had made evident short-term reductions in readmissions of older patients at risk and improved health outcomes [3–5]. Given that the Korean society reached the aging society, [6] and admissions to various healthcare levels increase over time, acquiring insights for care transition of the complex older population is essential.

Despite long-term care insurance (LTCI) mostly covers for home care and includes financial benefits for older adults in Korea who have chronic illnesses or disabilities, [7] many older adults are transferred to institutions such as rehabilitation hospitals, convalescent hospitals, or nursing homes, rather than discharged to their homes. The post-discharge period is often fragmented, and some patients miss out on golden time to recover their functional status through rehabilitation, putting a significant burden on the caregivers [8]. Current LTCI requires at least 6 months to operate the eligibility selection process. Therefore, older patients who were not in the insurance plan before hospitalization are challenged to receive the benefits as soon as they are discharged. The transitional care plan may play an essential role in matching optimal institutions to meet patient's specific care needs across medical, functional, and social domains. However, decisions on care transition are commonly deemed as a responsibility of patients and their family members, and largely performed in an ad-hoc manner, to date in Korea [9]. These unmet needs in care transitions may contribute to newly acquired disabilities and disability after hospitalization that is highly prevalent after inpatient care due to acute illness [10, 11].

The objective of this study is to explore barriers to meeting the needs of discharge planning for older adults in Korea. More specifically, by interviewing professionals working at tertiary hospitals in Seoul, Korea, we anticipated discovering barriers to transition care plans for inpatients with complex care needs and establishing effective solutions for the process. We also aimed to address specific concept which has to be built on our understanding of the current practice in the fields.

## Methods

### Study design

This is a qualitative descriptive designed study using focus groups. The study was conducted from September 2020 to November 2020 as an explorative study on improving care transition practice for patients with complex needs. To ensure the study quality, we considered parameters of the checklist by the Consolidated Criteria for REporting Qualitative research (COREQ) in performing interviews, analyzing data and reporting results [12].

### Sampling strategy

We used a purposeful selection strategy to identify participants. The interviewees were recommended by the Institute of Public Health and Medical Care, Seoul National University Hospital. Final 12 medical professional providers from 6 different institutions in Seoul were agreed to participate voluntarily. Among these 6 institutions, 7 participants came from the same academic institution and 5 were from public secondary or other tertiary hospitals. The focus group interviews were conducted by the moderator at the same time as both online and offline discussions. The participants received oral and written information about the study. The study protocol was reviewed and approved by the institutional review board of Asan Medical Center (2021–0679), that waived written informed consent.

### Data collection

A semi-structured interview guide was used including opening questions, introductory questions, transition questions and key questions. The focus group interview began by introducing participants with their current positions and specialties. After the introduction, opening question was proposed for an ice-breaker to encourage everyone to participate, then moved on to transition and key questions [13]. Interview questions were extracted from 2 pilot roundtable meetings with the participation of authors of this study on clinically encountered problems in care transition situations for older inpatients. Key questions discussed during the interview are listed in Table 1 and were proposed to elicit clinicians’ in-depth perspectives about the barriers to transferring older patients from general hospitals to community-based secondary or primary care.

**Table 1:** Focus-group interview key questionnaires

Category	Question
Participants’ Profile	Current position at the workplace
Barriers	Difficulties in discharge and transfer processes for older patients with complex care needs
	Patients’ perceptions on discharge and transfer
	What resource is required for optimal transfers?
	How can these difficulties be alleviated?

### Data analysis

The focus group interview discussions were audio recorded and transcribed verbatim. It was analyzed through 5 stages (familiarization, identifying a thematic framework, indexing, charting and mapping, and interpretation) of framework analysis [14]. Initially, the research team comprehensively read the written transcripts to devise the contents of responses by subjects. Then, respondents’ responses were indexed and categorized by key questions. The interpretation method is as follows: (1) Analysis of attitudes (positive/negative/neutral) for each participant. (2) Derivation of reasons and foundation for attitude. (3) Analysis of linkage or causal relationship with other topics. (4) Extended analysis of opinions or ideas. (5) Alteration level analysis of response contents. Thereafter, subdomains were constructed from participants' corresponding remarks, and barriers and solutions by these subdomains were largely grouped into patient and institutional factors.

## Results

### Participant characteristics

All of 12 Interviewees were attending physicians. 10 were affiliated with a tertiary hospital, and 2 were from public secondary hospitals. The mean age group was 30–39, and all had more than 2 years of experience in the current position. 8 out of 12 were women (Table 2).

**Table 2:** Characteristics of the interviewees

Category	Current Workplace / Department	Level of care	Current Position	Sex	Age Group	Work experience in current position
Online	Korean Red Cross Hospital / Internal Medicine	Secondary hospital	Attending physician	M	40–49	3-5y
Online	Seoul Seobuk Hospital / Family Medicine	Secondary hospital	Attending physician	F	40–49	3-5y
Online	National Medical Center / Internal Medicine	Tertiary hospital	Attending physician	F	30–39	< 3y
Online	Asan Medical Center / Division of Geriatrics	Academic/ Tertiary hospital	Attending physician	M	30–39	< 3y
Online	Seoul National University Hospital / Internal Medicine	Academic/ Tertiary hospital	Attending physician	M	30–39	> 5y
Offline	Seoul National University Hospital / Internal Medicine	Academic/ Tertiary hospital	Attending physician	F	30–39	> 5y
Offline	Seoul National University Hospital / Internal Medicine	Academic/ Tertiary hospital	Attending physician	F	30–39	> 5y
Offline	Seoul National University Hospital / Internal Medicine	Academic/ Tertiary hospital	Attending physician	F	30–39	> 5y
Offline	Seoul National University Hospital / Internal Medicine	Academic/ Tertiary hospital	Attending physician	F	30–39	> 5y
Offline	Seoul National University Hospital / Internal Medicine	Academic/ Tertiary hospital	Attending physician	F	30–39	> 5y
Offline	Seoul National University Hospital / Internal Medicine	Academic/ Tertiary hospital	Attending physician	F	30–39	> 5y
Letter	Seoul Medical Center/ Internal Medicine	Academic/ Tertiary hospital	Attending physician	M	40–49	> 5y

### Barriers and solutions to transition care

Using framework analysis concentrating on focus group interviews, we revealed two main domains of the barrier, which included multiple subdomains for each of them. The first domain was a patient factor barrier, a composite of (1) Incomprehension of the healthcare system; (2) Lack of communication with caregivers or decision makers; and (3) socioeconomic status; and (4) patient perception.

The second domain, institutional factors included: (1) different fee structures across the different levels of care; (2) high barrier to accessing health service in tertiary hospitals or to be referred to; (3) poor communication between institutions; and (4) insufficient subacute rehabilitation service across the country (Table 3).

**Table 3:** Barriers and Solutions to transferring older patients from tertiary-care to community-based healthcare system

Domain	Subdomain	Illustrative Quotations		
		Barrier	Solution	
Patient Factors	Patient-centered communication (Incomprehension of the healthcare system)	“There are many cases where patients believe that if they want to remain admitted, they could stay as long as they want in tertiary hospitals.” “They think transfer-out as an alternative to the difficulty of admission to a tertiary hospital. Some older patients wait for days at the ED for admission.” “Older patients do not have a good understanding of the healthcare system, especially they do not recognize that the functions of secondary or convalescent hospitals are different from those of advanced general hospitals. Dislike, resistance, or negative emotions are the first obstacles encountered when we decide or recommend transferring older patients to another hospital.”	“It is important to have sufficient communication about the treatment direction between the hospitals and the older patients or decision-makers.” “Necessary to form a consensus that tertiary hospital healthcare is no longer needed and provide promising continuity and integration of medical treatment through referrals.”	
	Lack of communication with caregivers or decision-makers	“Caregivers do not receive sufficient explanation about treatment direction” “When older patients are transferred into our palliative care wards, they often come without knowing what palliative care is. Therefore, a lot of times they want more active treatment”	“Prior to transfer, we need a consultation with the patients or caregivers. Therefore, we can reassure the treatment directions as well as the education for upcoming facilities.” “Multidisciplinary approach is needed to bring together a group of healthcare professionals from different fields within the institution, including nurses, social workers, public health cooperation managers, and doctors.”	
	Socioeconomic status	“Decision-making based on economic factors rather than medical factors causes another side effect.” “I have seen many older patients who do not want to be transferred out to higher-level hospitals because they would experience a much greater financial burden.”	“A transition care plan needs to be built in consideration of socioeconomic factors because of expensive caregiver fees, and to clearly outline how much the families or older patients are willing to pay for the facilities.”	
	Patient Perception	“Older patients with high anxiety do not accept that the current tertiary hospital treatment is no longer necessary.” “Prior experience of long ED stays or decline of admission due to lack of inpatients bed availability developed anxiety among older patients regarding the process of readmission in the future for possible clinical deterioration.”	“In order to relieve the anxiety of patients and their caregivers, a system that guarantees the continuity and integrity of treatment is required.” “Reinforcement of supportive care through patient-centered remarks in the medical records, medical referrals, and public medical teams was necessary.”	
Institutional Factors	Different Fee structure	“Each medical institution provides a different pricing system. Tertiary teaching hospitals adopt a fee-for-service payment model. However, secondary general hospitals and public healthcare are based on DRG payment to all patients.” “We cannot apply the identical prescriptions received from tertiary level hospitals. Patients complain and it hinders consistency and integrated treatment.”	“In convalescent hospitals, a transient fee-for-service scheme for transferred patients from upper-tier hospitals might be beneficial for more appropriate subacute care and rehabilitation.” “More rational fee schemes for older patients with varying medical- and functional care needs are needed.”	
	Barriers to accessing health service in tertiary hospitals/ Referrals	“It is really difficult to refer older patients back to tertiary care other than for emergency situations because of an inadequate number of hospitals [tertiary hospital] beds. Patients wait for days in the ED.”	“Better systems than the current phone call- or written note-based transfer inquiry model are required. Systems or routes to expedite reverse-transferring older patients who were recently transferred from tertiary level care, require higher-level treatment with deteriorating conditions in secondary hospitals or convalescent hospitals.” “Easy and usable ways of communications between doctors are needed.”	
	Insufficient cooperation and mutual communication between institutions	“When I refer a patient with a catheter, I need to know if the hospital is capable of managing such conditions.” “If I am referring a cancer patient who is on chemotherapy, I need to know transparently whether there is an oncologist who can manage the patient.” “Transparent information disclosure between institutions and standardized information management is required but it is difficult to know this when we transfer our patients.” “Important medical information is intentionally omitted. This leads to breaking the trust of the medical cooperation system.” “Basically, there is a difference in medical philosophy.” “Residents at the university hospital miss critical information in the patients’ medical records regarding what patients might need when they are transferred.” “After transferring the patients, no one follows up on what happened to the patient.”	“Transparent communication between physicians and educational institutions (including residents) to facilitate better communication.” “Standard formats and checklists in transfer or discharge records should be developed.” “Case management for patients with care transitions, on post-transfer issues.” “Opportunities to communicate with healthcare personnel in different care settings are needed to understand each other”	
	Need for subacute rehabilitation treatment services	“After the acute phase, patients often need rehabilitation. However, it is tough to transfer patients to legitimate rehabilitation facilities because we face a rehabilitation facility shortage “Rehabilitation is often undertaken in long-term nursing facilities, which reduces the quality of rehabilitation treatment itself.”		“Subacute care model for acutely admitted patients with complex care needs should be developed and implemented.” “More rehabilitation hospitals are needed. Otherwise, long-term hospitals (convalescent hospitals) should provide more patient-specific post-acute care, upgrading from the current minimal support for basic medical needs.”

*ED* Emergency department

Although the subject of the interview was focused on older adults, some barriers apply to non-older patients. However, we did not differentiate barriers by age group.

### Patient factors (Table 3)

#### Incomprehension of the healthcare system

A physician said: “…patients believe that if they want to remain admitted [in tertiary care], they could stay as long as they want.” Another participant noted, “Older patients do not have much understanding of the healthcare system; they do not recognize that the functions of secondary or convalescent hospitals differ from those of advanced general hospitals.” Dislike, resistance, or negative emotions were the first obstacle encountered when physicians decided to transfer a patient.

#### Lack of communication with caregivers or decision makers

Providers often confront mismatches between caregivers’ real needs and the available resources from facilities or vice versa. As one physician explained, “When patients were transferred into our palliative care wards, they often come without knowing what palliative care is…a lot of times they want more active treatment.” This is an example of a lack of communication. Providers noted that in the absence of a standardized assessment for the transition care plan and shared decision making, patients and caregivers might make wrong choices without knowing what they indeed want.

#### Socioeconomic status

Socioeconomic status was another commonly mentioned barrier. Participants noted that socioeconomic status could hinder patients' or caregivers’ decision-making. For example, caregiver fees or financial burden due to high-level hospital care were associated with both ways of transfers toward either lower or higher tier level of care. One said, “Decision-making based on economic factors rather than medical factors causes another side effect.” Most clinicians acknowledge that disease-specific care is not the best way to make decisions for older patients; however “in reality, it is difficult to consider individual’s financial capacity, social factors, priorities in healthcare, and treatment burden all at the same time.”

#### Patient perception

Physicians indicated that older patients’ perceptions of the discharge process could become a barrier to create a smooth transition of care. A provider cited that “older patients with high anxiety do not accept that the current tertiary hospital treatment is no longer necessary”, and another clinician mentioned that “Prior experience of long ED stays or decline of admission due to lack of inpatients bed availability had built anxiety on the process of readmission in the future for possible clinical deterioration.” How older patients perceive the discharge process in the past influenced the anxiety level and was deemed to have negative insights on transitions.

### Solution for patient factors

All physicians who participated in the interview had experienced negative feedback from patients and caregivers when transferring older patients after acute treatment. Based on our findings of these barriers, we discussed solutions to alleviate the struggles. Almost all participants suggested having sufficient communication and empathy for making patient-centered decisions for the future directions to avoid conflicting recommendations and further treatment burdens. Participants cited the multidisciplinary approach to transition care to mitigate the communication failure, with well-defined responsibilities from supplementary resources within the hospital. Physicians also emphasized the importance of preceding consultation about the institution to be transferred, prioritizing care preferences, and confirming whether it meets the needs of the patient and the caregivers. In addition, one provider cited, “in order to relieve the anxiety of older adult patients and their caregivers, a system that guarantees the continuity and integrity of treatment is required. Reinforcement of supportive care through patient-centered remarks in the medical records, medical referrals, and public medical teams can be a solution for more integrated care after transitions.

### Institutional factors (Table 3)

#### Different fee structure

South Korea introduced national health insurance in 1977 and adopted a nationwide fee-for service (FFS) system. In the beginning of 2012, the Korean government mandated to participate diagnosis-related groups (DRGs) payment system for several diseases in smaller hospitals and expanded to all medical institutes except long-term care hospitals and some public hospitals. “When a cancer patient with ascites who are continuously receiving 20% albumin twice a day, which is not subject to reimbursement coverage, for non-reimbursements with patient consent in tertiary hospitals, is transferred to DRG-applied hospital, the secondary hospitals or public hospitals cannot pay the cost even with non-reimbursements payment, so the treatment cannot be continued.”

#### High barrier to accessing health service in tertiary hospitals or to be referred to

Older patients in long-term care hospitals are at greater risk for acute illness. However, they have a low referral rate back to tertiary care hospitals. Participant cited, “It is really difficult to refer older patients back to tertiary care, other than emergencies due to an inadequate number of hospitals [tertiary hospital] beds. Patients wait days in the ED.” After being transferred out from tertiary care, many older patients usually stay in long-term care facilities with higher tertiary care accessibility.

#### Poor communication between Institutions

Every participant in the interview mentioned the communication barrier between different levels of care and institutions. For example, the provider noted, “When I refer a patient with a catheter, I need to know if the hospital is capable of managing such applications… but many times I do not know.” Another participant mentioned tertiary care side of the communication drawback: “Residents (or attending physicians) at university hospital miss critical information regarding what patients might need when they inscribe their medical records.”

#### In need of subacute rehabilitation treatment service

A Physician cited, “After the acute phase, older patients often need rehabilitation. Because we are facing rehabilitation facility shortage, it is really hard to transfer older patients to legitimate rehabilitation or subacute care facility.” Long term care hospitals (LTCH, convalescent hospitals) are bound to daily fixed fee schemes upon patient levels. Under current fee structure, convalescent hospital is rarely able to provide either sub-acute care for complex medical problems or rehabilitation for disabilities associated with acute care. Participants noted that, in many cases, there is no place to discharge or transfer older patients requiring treatments with intravenous medications or fluids and physician’s detailed attention to ‘soft-land’ acute high-level treatment.

### Solutions for institutional factors

In the interview, participants agreed that current care delivery system and fee structure of Korea precludes effective care transitions of older patients with diverse care needs. For effective subacute care and rehabilitation, everyone supported more patient-centered fee structures other than current setting-dependent fee schemes that reinforce the formation of healthcare silos. Also, participants noted that better education for physicians including residents on patients’ medical, functional, psychosocial needs are overarchingly needed to alleviate problems arising from disease-centric, one-size fits all discharge planning. For improved communications, physicians agreed that standard formats and checklists in transfer or discharge records could be helpful. To alleviate long waiting in the emergency department for unexpected medical issues after a transfer, participants acknowledged that patient-centered longitudinal case management for transition care also enables reverse transfers to initial higher tier centers without going through emergency department that is often very burdensome to both patients and caregivers.

## Discussion

Despite advancements in medical treatments in Korea, management of older patients after discharge remains fragmented without specific transitional care plan. To improve the continuity of care of older patients, comprehensive understanding of current barriers through objective discussions were crucial. In this study, we found that barriers to establishing transition care planning in hospitalized patients with complex care needs can be largely classified into 2 domains: patient and institutional. We also recognized that these barriers might be alleviated by effective communication strategies and patient-centered care models accounting for functional and medical issues [15]. These findings contrast with previous government-driven care transition models that primarily focus on medical resources and hospital networks rather than patient-centric issues impeding effective transitional care.

The fragmentation in the transitional care process derives from the setting of disease-centered practiced culture since there was no defined universal approach. Lack of communication comes from treatment-focused and careless attitudes towards what would essentially matter to the patient. For patient-centered approaches, we may adopt frameworks of the Age-Friendly Health Systems initiative that led by The John A. Hartford Foundation and the Institute for Healthcare Improvement in partnership with the American Hospital Association and the Catholic health Association of the United States [16]. The goal of the system is to making U.S. health care systems age-friendly across all care settings through implementation of the 4 Ms framework: What matters; Medications; Mobility; and Mentation [16, 17]. The concept values the extent to what really matters to the older adult and their families, unlikely to widespread disease-centered healthcare system. Traditional provider-driven approach cannot facilitate unprecedented population ageing wherein complex and interrelated needs are detected. By embracing the patient-centered care and successfully implemented the 4Ms, many health systems in the U.S improved patient satisfaction, family engagement, length of stay and readmissions [15]. This being said, Age-Friendly health care is little known and practiced in Korea compared to its rapid spread of the framework in the U.S. hospitals and medical practices. Through an active adoption of patient-centered care with 4Ms framework, traditional provider-driven approaches in Korea can be mitigated and bring a more sophisticated stepwise transition to a long-term care for older patients.

From the discussions in our study, we were able to address information transfer deficits between hospitals were another common barrier to effective transitional care. This does not apply only to older patients but also to general patients. In addition, patients’ misperceptions of healthcare across the primary care system and low chance of being referred to tertiary hospitals escalate the level of anxiety in patients after being discharged from tertiary care hospitals. It is crucial to recognize Korea's current care delivery system to understand this problem. Although healthcare coverage in Korea had achieved almost nationwide as of today, [18] the role of primary care is not yet identified, and patients are lacking with their key case managers [19]. Patients in Korea can choose any type of outpatient hospital clinic simply without having referrals from primary care physicians, even to hospital-level institutions for their first visit [19, 20]. This creates a competitive relationship between hospitals, rather than being cooperative. Because of the unique system, patients prefer larger hospitals over small clinics in the community for primary care [21, 22]. Under this circumstance, primary care has been devalued in Korea and failed to establish a sustained primary care physician(PCP) and a patient relationship that prolongs patients’ overall medical concerns over time and events in between [23]. It is often the patient’s role to communicate one’s medical concern with hospitals through medical documents, and therefore it carries communication hardship. One study applied PCP-Enhanced Discharge Communication Intervention which decreased post-discharge medication discrepancies [24] indicating the importance of PCP involvement in the hospital discharge process as patient-centered case managers [25]. Older patients at high risk of hospitalization or otherwise in need of complex treatments can ensure continuity of care by providing close follow-up with the PCP in the primary care setting. In addition, this guarantee of continuity of care can alleviate communication difficulties and sharing of medical information between hospitals [25, 26].

Following discharge for older adults after an acute illness in tertiary institutions, many older adults who have difficulties in activities of daily living are transferred to LTCH, which is a unique form of long-term care (LTC) in Korea [7]. LTCHs are widespread in Korea. Still, its role has not been distinguished from nursing homes unlike its first intention, where the major priority was functional rehabilitation to return them home with greater independencies [27]. A study by Kim et al. concluded that the current LTC system in Korea should be redesigned as a person-centered delivery through integrated assessment system; therefore the service can address both health and social care needs [28]. Other studies showed that functional decline that is associated with hospitalization is highly prevalent in older adults and recovery of function is critically important [29–31]. Rehabilitation centers in Korea are likely to have specific priorities to specific neurological or orthopedic insults, not a functional deconditioning related to general medical events, with specific fee structures for indicated conditions. Therefore, rehabilitation facilities usually cannot accept patients with disabilities due to frailty or deconditioning after acute illness. Even though the Pilot Project of Rehabilitation Medical Institutions was started in 2020 to resolve this gap, only 45 institutes have participated the program to date. Therefore, many patients with mixed medical and functional requirements are frequently transferred to LTCH, while the provision of optimal subacute care and rehabilitation is highly unlikely due to the current daily fixed fee scheme. Similarly, under the DRG fee structure, appropriate subacute care for patients from tertiary hospitals was unrealistic due to economic losses. In our study, participants urged that patient-centric fee structure in peri-transition care situations is imperative to resolve barriers to transferring complex patients from tertiary hospitals and may also help alleviate current ‘patients’ inclination’ toward large centers.

Concern over the healthcare silo effect and poor cooperation between institutions has been growing, leading physicians in practice difficult to position patients at appropriate places. Physicians in the study noted that hospitals receiving older patient transfers in the community must be transparent to what extent they can manage patients. Another significant factor is deficits in information between hospitals. Discharge summaries and discharge letters often miss substantial and essential patient information [32] few data is passed over for the healthcare continuum. Previous studies found that proposed discharge date and destination collection from patients with interdisciplinary collaboration teams, raised the perceptions of patients’ awareness of discharge plans, prevented unnecessary delays in discharge, and provided physicians about alternative destinations regarding older patients’ preferences [33]. Another aspect to consider in Korea, is to utilize nursing communication. Discharge summaries in Korea are written by medical doctors yet nursing or other professional’s comments are not included. However, multidisciplinary professionals are involved in providing patient care during the hospitalization, particularly nurses are the closest staff members who could suggest persistence of problems and difficulties especially for older adult’s future care. Implementing multidisciplinary components in discharge summaries; including nursing, physical therapy, and social worker for post-discharge care plan would improve breakdown in communication [34, 35].

Our study has several limitations. This is qualitative study and only included physicians from upper-level healthcare hospitals, or academically affiliated in urban area. The population may not be generalizable to other settings. However, we identified a wide range of barriers consistent with smaller institutions and discussed the issues that are related to primary care settings. Also, ongoing qualitative and quantitative studies with the involvement of multidisciplinary professionals would potentially address this limitation. Second, there was only one focus group for our study. Multiple focus group interviews may have helped to reach adequate saturation of the data. Furthermore, it would have been interesting to look at how the patients or caregivers’ perspectives differ from healthcare professionals in transition care planning. As yet, there is no study conducted to evaluate barriers on a particular topic in Korea. Therefore, the study is unique and has several strengths. Interviewing acute healthcare professionals who are in part of the decision making for the discharge planning may have allowed us to better understand what causes disruptions continuity of care for patients. Second, the qualitative research data could give detailed and rich real-world information about what has been experienced from each participant that were able to reveal the complexities what is being studied. Further research is needed on health systems considering policies that supports the topic with interventions.

## Conclusions

In this study, 12 medical professionals identified barriers to optimal transitional care plans, including patient’s and institutional factors. Older patients face more challenges arising from transitional care fragmentation, given that the complexity of functional care needs is additional. More patient-centered decision-making should be implemented to address current unmet care needs for patient discharge planning. Improved care structures for a peri-transition period may improve medical and functional outcomes after acute hospitalizations.

## Data Availability

The dataset used during the current study are available from the corresponding author on reasonable request.

## Abbreviations

ED: Emergency department
FFS: Fee-for-service (FFS)
DRG: Diagnosis-related groups
LTCH: Long term care hospitals
PCP: Primary care physician
